# Transcriptome profiling at osmotic and ionic phases of salt stress response in bread wheat uncovers trait-specific candidate genes

**DOI:** 10.1186/s12870-020-02616-9

**Published:** 2020-09-16

**Authors:** Diana Duarte-Delgado, Said Dadshani, Heiko Schoof, Benedict C. Oyiga, Michael Schneider, Boby Mathew, Jens Léon, Agim Ballvora

**Affiliations:** 1 INRES-Plant Breeding, University of Bonn, Bonn, Germany; 2 INRES-Crop Bioinformatics, University of Bonn, Bonn, Germany

**Keywords:** Bread wheat, Salt stress, Ionic stress, Osmotic stress, Comparative transcriptomics, QTL dissection

## Abstract

**Background:**

Bread wheat is one of the most important crops for the human diet, but the increasing soil salinization is causing yield reductions worldwide. Improving salt stress tolerance in wheat requires the elucidation of the mechanistic basis of plant response to this abiotic stress factor. Although several studies have been performed to analyze wheat adaptation to salt stress, there are still some gaps to fully understand the molecular mechanisms from initial signal perception to the onset of responsive tolerance pathways. The main objective of this study is to exploit the dynamic salt stress transcriptome in underlying QTL regions to uncover candidate genes controlling salt stress tolerance in bread wheat. The massive analysis of 3′-ends sequencing protocol was used to analyze leave samples at osmotic and ionic phases. Afterward, stress-responsive genes overlapping QTL for salt stress-related traits in two mapping populations were identified.

**Results:**

Among the over-represented salt-responsive gene categories, the early up-regulation of calcium-binding and cell wall synthesis genes found in the tolerant genotype are presumably strategies to cope with the salt-related osmotic stress. On the other hand, the down-regulation of photosynthesis-related and calcium-binding genes, and the increased oxidative stress response in the susceptible genotype are linked with the greater photosynthesis inhibition at the osmotic phase. The specific up-regulation of some ABC transporters and Na^+^/Ca^2+^ exchangers in the tolerant genotype at the ionic stage indicates their involvement in mechanisms of sodium exclusion and homeostasis. Moreover, genes related to protein synthesis and breakdown were identified at both stress phases. Based on the linkage disequilibrium blocks, salt-responsive genes within QTL intervals were identified as potential components operating in pathways leading to salt stress tolerance. Furthermore, this study conferred evidence of novel regions with transcription in bread wheat.

**Conclusion:**

The dynamic transcriptome analysis allowed the comparison of osmotic and ionic phases of the salt stress response and gave insights into key molecular mechanisms involved in the salt stress adaptation of contrasting bread wheat genotypes. The leveraging of the highly contiguous chromosome-level reference genome sequence assembly facilitated the QTL dissection by targeting novel candidate genes for salt tolerance.

## Background

Bread wheat (*Triticum aestivum* L.) is a major staple crop for global food security whose production needs to be increased by 60% to feed the world population by 2050 [1, 2]. Therefore, breeding programs should emphasize the genetic improvement of complex traits to increase yield potential under growth-limiting conditions [3]. The genetic studies of complex traits in wheat are challenging because it is an allohexapolyploid species containing three subgenomes (AABBDD) with highly repetitive DNA sequences (85%) and a total genome size of 16 Gb [4, 5]. Efforts from the International Wheat Genome Sequencing Consortium (IWGSC) have resulted in the release of a fully annotated and highly contiguous chromosome-level assembly sequence draft of the Chinese Spring cultivar that represents 94% of the whole genome [5–7].

The daily salt-induced degradation of 2000 ha of arable soil worldwide is a serious threat to global food security [8]. Among all the abiotic stress factors, soil salinity can cause significant yield reductions and decreased grain quality in wheat [9]. The salt stress adaptation response is a complex trait because it affects the coordinated action of gene networks in several metabolic pathways causing changes in crucial physiological processes [10, 11]. Therefore, the targeting of candidate genes for stress-related traits can be exploited in breeding programs to develop cultivars with increased salinity tolerance [12].

High salinity leads to physiological drought conditions, causes ion toxicity and cell oxidative damage that affect the plant growth [10, 11]. The plant growth response to salinity comprises two phases. The first corresponds to the osmotic phase, which is independent of the sodium accumulation in tissues. The rapid and often transient impact on plant growth in this phase is attributed to the osmotic effect of the salts in the rhizosphere because of reduced water potential [13–15]. Consequently, the osmotic stress-tolerant plants can adapt to the drought aspect of the stress through the maintenance of the stomatal conductance and the leaf turgor [16]. The early signaling events in the osmotic phase occur within seconds to hours after salt stress exposure and are crucial for the acclimation response of the plants [15]. A model proposes that in the osmotic phase, the root senses salt stress and second messengers as reactive oxygen species (ROS) and Ca^2+^ are spread as signals to the aerial parts. These signals trigger adaptive responses to cope with the Na^+^ ions that reach photosynthetic tissues and cause toxic effects in the following stress phase [17]. Second, the ionic phase continues as a result of salt accumulation in leaves and takes days or weeks to manifest. In this phase, the senescence of older leaves is caused by the plant’s inability to tolerate the toxic concentrations of salts in the tissues [13, 18]. To reduce the toxicity effects, tolerant plants can excrete the Na^+^ accumulated in shoots by the roots or compartmentalize Na^+^ and Cl^−^ in vacuoles to avoid toxic concentrations in the cytoplasm [19, 20]. The limiting effect of salinity on crop productivity in both stress phases is mainly due to its effect on the photosynthetic process, which results in a substantial decrease in biomass accumulation [21, 22].

The genetic mapping studies have allowed the detection of statistical associations of molecular markers with phenotypic values of a quantitative trait to find locations of quantitative trait loci (QTL) in the genome of a given species [23, 24]. Several mapping analyses in bread wheat have identified QTL with effect on salt stress-related traits [25–29]. Because of the limited mapping resolution of most of these studies, each QTL interval contains many potential quantitative trait genes (QTGs) influencing the trait variation. Studies that combine genetic mapping and transcriptomic analyses can significantly reduce the number of these genes and provide strong candidate QTGs for the identified QTL following the assumption that natural genetic variation can underlie complex traits by regulating gene expression mechanisms [24, 30–32].

The Next-Generation Sequencing platforms comprise rapidly evolving high throughput methods that are selected to produce new insights into the genome, transcriptome and epigenome of plants to assist the breeders in understanding the biological function of the genes [33]. Transcriptomics studies allow the identification of pathways that are regulated at the osmotic and ionic phases. A time-course transcriptomic analysis can be exploited to identify transcriptional changes during key physiological variations occurring in the salt stress response [34–36]. The Massive Analysis of cDNA 3′-ends (MACE) sequencing protocol is an alternative to regular RNA-sequencing, where a single sequence fragment represents one transcript [37]. Therefore, the output from this protocol is digital and strand-specific, facilitating and increasing the accuracy of expression quantification. Furthermore, this method may provide high resolution to detect genes with low or moderate expression levels and short transcripts [37, 38]. These features of MACE contribute to explore the complex wheat transcriptome with less sequencing data and refine gene models towards the 3′-ends [39].

The main goal of our research was to pinpoint candidate genes operating in salt stress response pathways leveraging the recent annotated and highly contiguous chromosome-level genome sequence assembly from bread wheat [5]. For that, the MACE approach was used for dynamic transcriptome profiling at osmotic and ionic phases. A comparative transcriptomic analysis across diverse genetic backgrounds and stress phases allowed the identification of core and differential salt-responsive gene categories. Some of these categories were linked with photosynthesis measurements during the osmotic phase and K^+^ and Na^+^ accumulation studies performed at the ionic stage. The stress-responsive genes located within QTL for salt stress-related traits were targeted as candidate genes controlling trait variation. This study contributes to QTL dissection by providing candidate genes for further functional analyses to validate them as breeding targets.

## Results

### Leave transcriptome sequencing at osmotic and ionic phases of salt stress

**Fig. 1:**
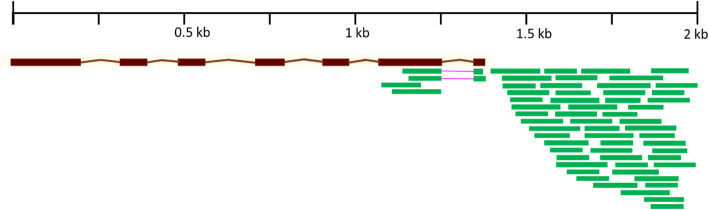
Representation of the prolonged 3′-end of the gene *TraesCS7D02G051200* with reads (in green) mapping beyond the gene model

The MACE protocol was used to compare the levels of expression of genes in the contrasting genotypes Zentos (salt-tolerant) and Syn86 (salt-susceptible) at the osmotic phase and Altay2000 (salt-tolerant) and Bobur (salt-susceptible) at the ionic stage. Salt-responsive genes were identified at 8, 15, 30 min and 4 h after stress exposure (ASE) in the osmotic phase and 11 and 24 days ASE in the ionic stage. The sequenced libraries originated from four pooled plants in each control and stress time point and the reads were aligned to the reference genome. The libraries from Altay2000 and Bobur contained a higher number of total and duplicated reads than the libraries from the genotypes studied at the osmotic phase (Table 1). The exclusion of fewer reads after the quality control filtering and a greater mapping efficiency were observed in the ionic stress libraries compared to those from the osmotic stress experiment (Table 1). The details of reads processing and reference genome mapping from each library are included in the Additional file 1. From the total unique mapped reads, 86% were scored with the reference annotation, while 91% with the extended gene models. Therefore, with the extended annotation ca. 6 million of additional reads were detected in 12,019 genes listed in the Additional file 2. These genes accounted for 4.5% of the gene models predicted in the RefSeq v1.1 genome annotation [5]. The Fig. 1 exemplifies the prolonged 3′-end of the gene *TraesCS7D02G051200* with reads mapping beyond the defined gene model.

**Table 1:** Summary of MACE libraries processing and reference genome mapping (mean ± standard deviation)

	Osmotic phase	Ionic phase
**Libraries**	14	8
**Total millions of reads**	5.0 ± 0.7	9.0 ± 2.4
**Reads excluded after QC**^a^ **(%)**	7.8 ± 1.6	2.6 ± 0.3
**Mapping efficiency (%)**	83.6 ± 1.2	93.0 ± 0.3
**Millions of mapped reads**	3.8 ± 0.5	8.2 ± 2.2
**Multiple aligned reads (%)**	21.7 ± 3.3	19.7 ± 1.7
**Millions of unique mapped reads**	3.0 ± 0.4	6.6 ± 1.8
**Reads after deduplication (%)**	62.2 ± 3.7	53.9 ± 3.2

^a^QC: Quality control

### Identification of salt-responsive genes

To compare the level of expression of genes in response to salt stress, GFOLD (generalized fold change) values > 1 or < − 1 with parameter c = 0.01 were considered for the identification of salt-responsive genes at the time points from the osmotic and ionic phases [40]. The expression densities of the libraries from the four genotypes were compared and shown in the Additional file 3. The libraries from Syn86 and Zentos were comparable, as evidenced by their overlapping expression densities. Differently, a greater mean of the expression values was observed at 24 days ASE compared to the mean in the other time points (Additional file 3). This type of distribution of the expression is an indicator of high levels of PCR duplication of reads. Therefore, the deduplicated alignment files were used for the differential expression analysis at the ionic phase. After deduplication, the density plots revealed a better homogeneity of Altay2000 and Bobur samples (Additional file 3).

The differential expression analysis showed a reduced variability among genotypes (mean ± standard deviation) concerning the percentage of identified novel transcripts (4.5 ± 0.4%), low confidence (LC) (5.0 ± 0.8%) and high confidence (HC) (90.5 ± 1.1%) gene models. The D subgenome contained the highest percentage of salt-responsive genes (35.8 ± 1.7%) followed by B (31.4 ± 1.1%) and A (31.3 ± 1.5%) and unplaced superscafolds (1.5 ± 0.3%). The genome coordinates of novel transcripts are shown in the Additional file 4, and the expression levels of all salt-responsive genes are listed in the Additional file 5.

### Comparative analysis of the osmotic stress response

**Fig. 2:**
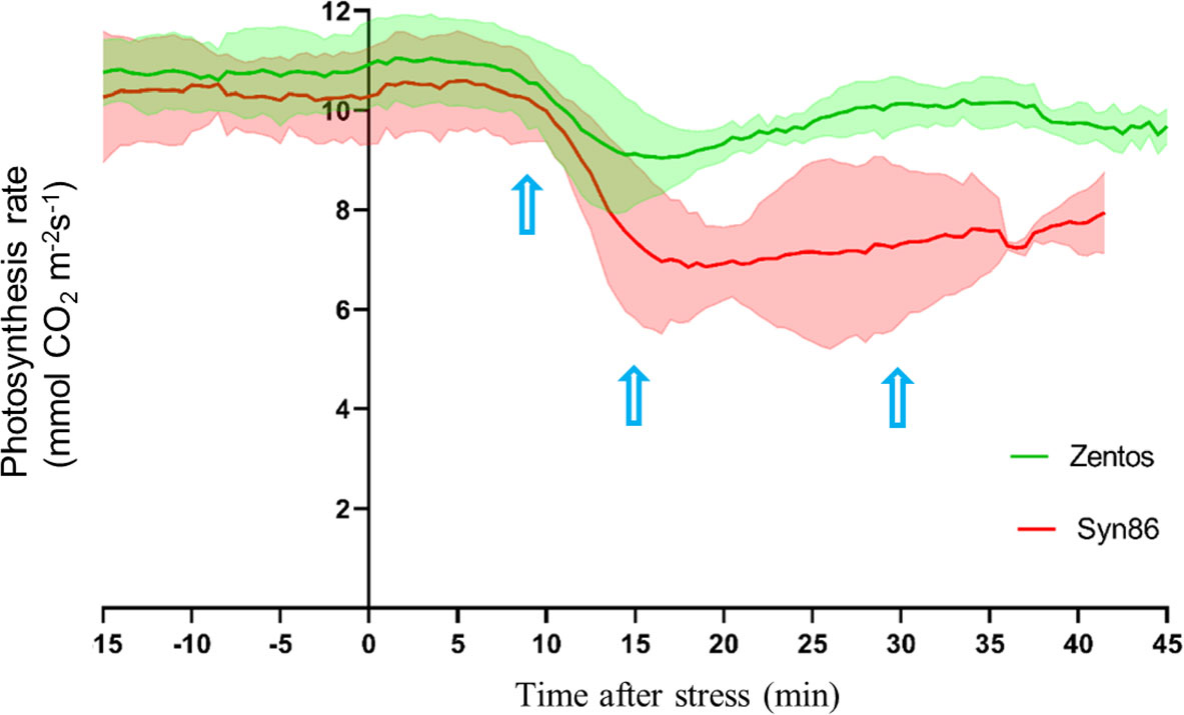
Photosynthesis rate curve of contrasting wheat genotypes studied during the osmotic phase adapted from Dadshani [27]. The shadows represent the standard deviation of the measurements, and the time points selected for the transcriptomic analysis are highlighted with blue arrows

**Fig. 3:**
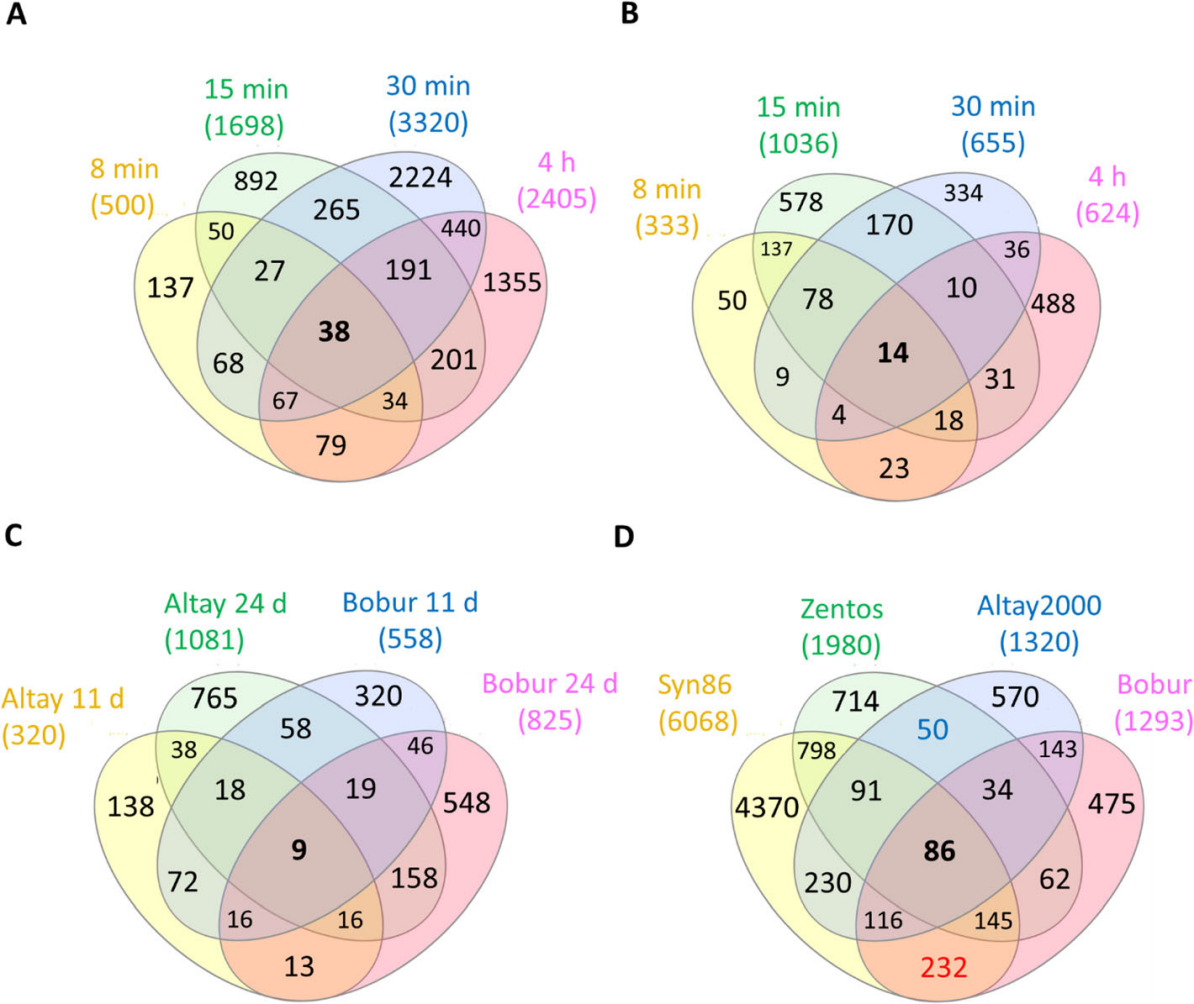
Venn diagrams of the salt-responsive genes in the contrasting genotypes studied. The total number of genes in each genotype and/or time point are shown above each diagram. (A) Diagram of the salt-responsive genes in Syn86 by time point; (B) diagram of the salt-responsive genes in Zentos by time point; (C) diagram of the salt-responsive genes in the two sampled days from the ionic stress phase; (D) diagram with the four genotypes. The blue number represents the genes shared by the tolerant genotypes, while the red number indicates the genes shared by the salt-susceptible

**Fig. 4:**
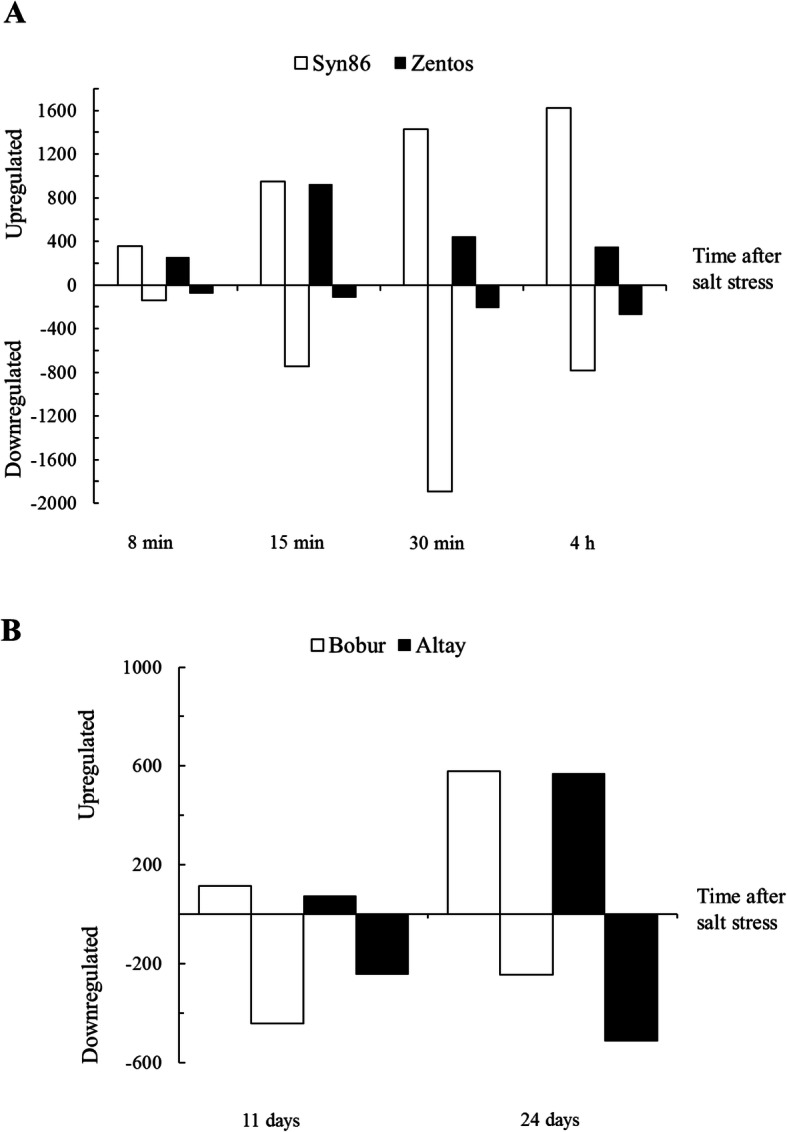
Distribution of up- and down-regulated salt-responsive genes across stress time points. (A) Osmotic phase and (B) ionic phase time points

A transcriptome profiling at the osmotic stress phase was performed to study the early plant reaction to salt exposure. The selection of time points for this analysis was based on the time course of the photosynthesis response under salt stress (Fig. 2) [27]. The greatest number of salt-responsive genes was observed at 30 min in Syn86 and at 15 min ASE in Zentos, whereas the lowest number was identified at 8 min ASE in both genotypes (Fig. 3a, b). Thirty-eight and 14 genes were differentially expressed simultaneously across all the time points in Syn86 and Zentos, respectively (Fig. 3a, b). The distribution of up and down-regulated genes revealed that Zentos had the highest number of up- and down-regulated genes at 15 min and 4 h ASE, respectively (Fig. 4a). In contrast, Syn86 had the highest number of up-regulated genes at 4 h and down-regulated at 30 min. In total, Zentos showed 75% of up-regulated genes, while Syn86 had 60%.

**Fig. 5:**
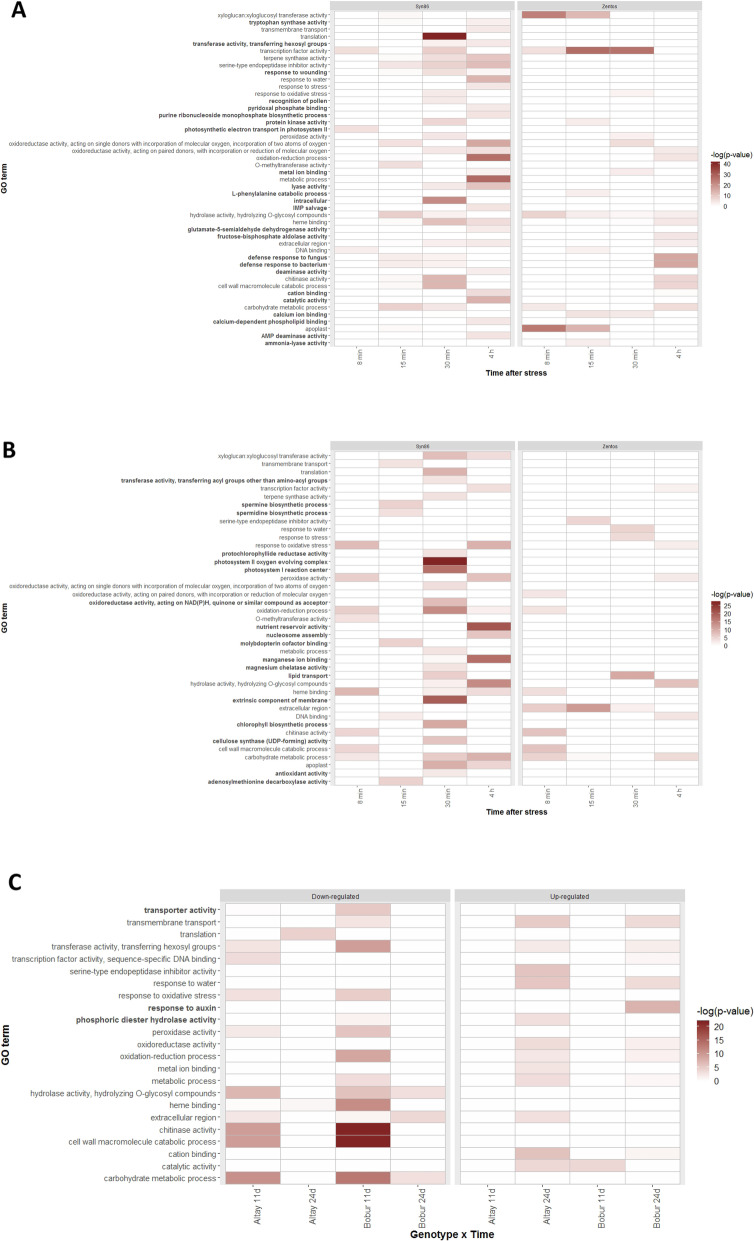
GO terms over-represented during the salt stress response. (A) Up-regulated and (B) down-regulated categories identified in the four stress time points sampled during the osmotic phase; (C) up- and down-regulated categories observed in the two stress time points from the ionic phase. Bold ontologies are categories specific for each heatmap. The –log_10_ transformation of the corrected *p*-values highlights the categories with greater significance that are therefore better over-represented. Transformed values > 3 are significant (corrected p-value < 0.001)

Over-represented gene categories in each time point of stress were revealed by the gene ontology (GO) enrichment analysis. Highlighted in the heatmaps (Fig. 5a, b) are the 24 and 18 over-represented ontology terms that were exclusively up- and down-regulated, respectively. Among the up-regulated categories, genes involved in response to wounding and tryptophan synthase activity were over-represented in the susceptible genotype, whereas in Zentos were genes with calcium-binding domains. Defense response to fungus and bacterium, transcription factor activity and protein kinase coding genes were over-represented and up-regulated in both genotypes (Fig. 5a). The over-representation of spermine and spermidine biosynthesis and antioxidant activity genes was identified in the down-regulated categories from Syn86 (Fig. 5b). The Additional file 6 details for each over-represented category the total number of genes in the background, the number of salt-responsive genes assigned to the term (GA), the number of expected genes (GE) and the fold change. The latter resulted from GA divided by GE and indicated how high each significant enrichment was.

Five clusters with particular expression profiles included 96% of the salt-responsive genes from each genotype and are shown in the Additional file 7. According to the expression tendency, the primary cluster of Zentos contained genes up-regulated at 15 min followed by a cluster of genes up-regulated at 30 min, while genes down- and up-regulated at 30 min corresponded to the two major clusters from Syn86. These clusters contained 54.5 and 57.3% of the total salt-responsive genes from Zentos and Syn86, respectively. Additional file 8 lists the GO terms over-represented in the clusters. These terms coincided mostly with the categories over-represented in the time points that showed greater magnitude in the expression profiles (Fig. 5a, b).

### Time course of gene expression and photosynthesis rate during the osmotic phase

**Fig. 6:**
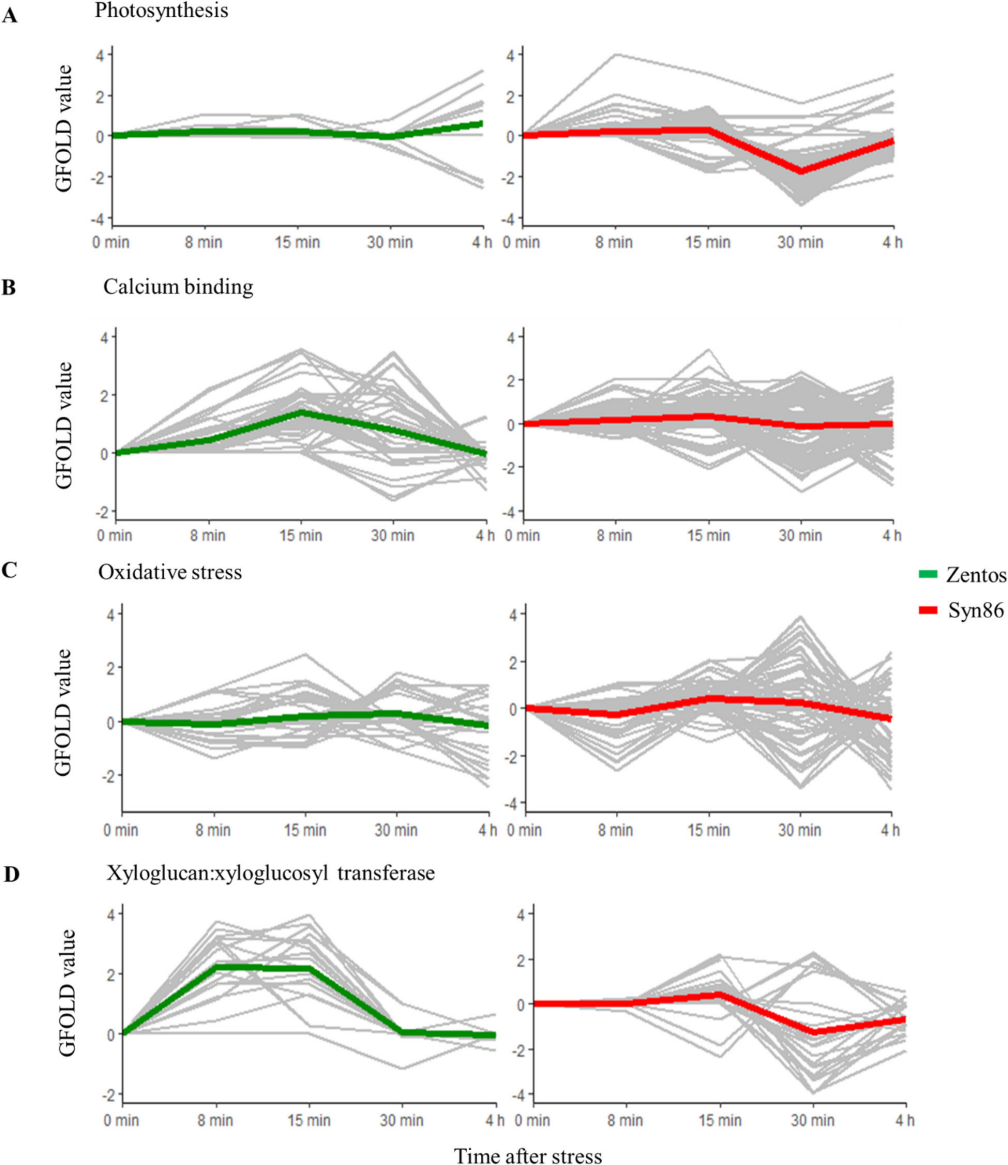
Generalized fold change (GFOLD) values of the time course relative expression of selected gene ontologies in the contrasting genotypes at the osmotic phase. In each expression profile frame, the gray lines show the time course expression pattern of each gene, and the red or green lines are LOESS (locally estimated scatterplot smoothing) curves that represent the expression tendency of the clusters of genes

The expression profiles of some over-represented gene categories relevant to the osmotic phase showed crucial differences in the two genotypes. Therefore, a comparison of the expression profiles of the photosynthesis-related, calcium-binding, oxidative stress response and xyloglucan:xyloglucosyl transferase activity genes was performed (Fig. 6). The over-representation of the photosynthesis category was observed in the susceptible genotype with 101 genes, whereas only 11 transcripts from this term were salt-responsive in Zentos (corrected *p*-value > 0.001) (Fig. 6a). The up-regulation of eight genes related to electron transport in photosystem II (PSII) was observed at 8 min ASE when the photosynthesis rate starts to decrease in Syn86 (Fig. 2). At 30 min, when the photosynthesis rate showed recovery but was still inhibited (Fig. 2), 91 transcripts from photosystems I and II categories were down-regulated with relative expression values ranging from − 1.1 to − 3.4 (Fig. 6a). In the case of Zentos, the few photosynthesis genes affected by salt stress were observed at 4 h ASE (Fig. 6a), which agreed with the greater photosynthesis stability detected in the first minutes ASE in this genotype (Fig. 2).

The locally estimated scatterplot smoothing (LOESS) curve from the 50 salt-responsive calcium-binding genes of the tolerant genotype revealed a gene up-regulation tendency at 15 min. Thirty-four transcripts were identified in this time point with relative expression values ranging from 1.0 to 3.4 (Fig. 6b). From these genes, 32 contained an EF-hand calcium-binding domain. This term was not over-represented in Syn86 (corrected *p*-value > 0.001) even though more genes showed differential expression than in Zentos. Thus, Syn86 presented various expression patterns of 129 calcium binding genes. Most of these genes (40) were down-regulated at 30 min with GFOLD values ranging from − 1.0 to − 3.1 (Fig. 6b). The majority of them (29 genes) were components of the oxygen-evolving complex from the PSII [39]. This result was also in line with the suppressed photosynthesis rate of Syn86 at this time point (Fig. 2). Other genes from this category were up-regulated in this genotype, 35 at 30 min and 21 at 15 min ASE.

From the oxidative stress response category, 33 salt-responsive genes were identified in Zentos. Eight and ten of them were up-regulated and showed relative expression values lower than 2.5 at 15 and 30 min ASE, respectively. The down-regulation of eight genes was observed at 4 h with expression values ranging from − 1.0 to − 2.4 (Fig. 6c). In contrast, 59 genes in Syn86 displayed diverse expression patterns with higher relative expression values than Zentos (Fig. 6c). The expression values from the down-regulated transcripts fluctuated from − 1.0 to − 3.5, and the up-regulated genes revealed a GFOLD value range from 1.0 to 4.2. The highest number of salt-responsive oxidative stress genes was observed at 30 min (38 transcripts) with both up- and down-regulated transcripts included. These greater transcriptional variations in the susceptible genotype were congruent with the inhibited photosynthetic activity observed at this time point (Fig. 2).

Finally, all the salt-responsive cell wall genes corresponded to the xyloglucan:xyloglucosyl transferase activity category. Eighteen genes were identified in Zentos, from which 14 showed up-regulation at 8 and 15 min with GFOLD values ranging from 1.1 to 4.0 (Fig. 6d). In Syn86, 24 genes from this category were salt-responsive. The LOESS curve highlighted the down-regulation of 16 transcripts at 30 min with relative expression values ranging from − 1.0 to − 4.3 (Fig. 6d).

### Comparative analysis of the ionic stress response

To better understand the later phase of plant reaction to salt exposure, a comparative transcription profiling at the ionic stress phase was performed. This analysis revealed the fewest transcriptional changes in Altay2000 at 11 days (Fig. 3c). The simultaneous differential expression of nine genes was identified across genotypes and time points (Fig. 3c). At 24 days ASE, more genes were up-regulated than down-regulated, whereas the opposite pattern with more down-regulated genes was found at 11 days ASE in both genotypes (Fig. 4b). In total, Altay2000 and Bobur contained 54 and 50% of down-regulated genes, respectively.

**Fig. 7:**
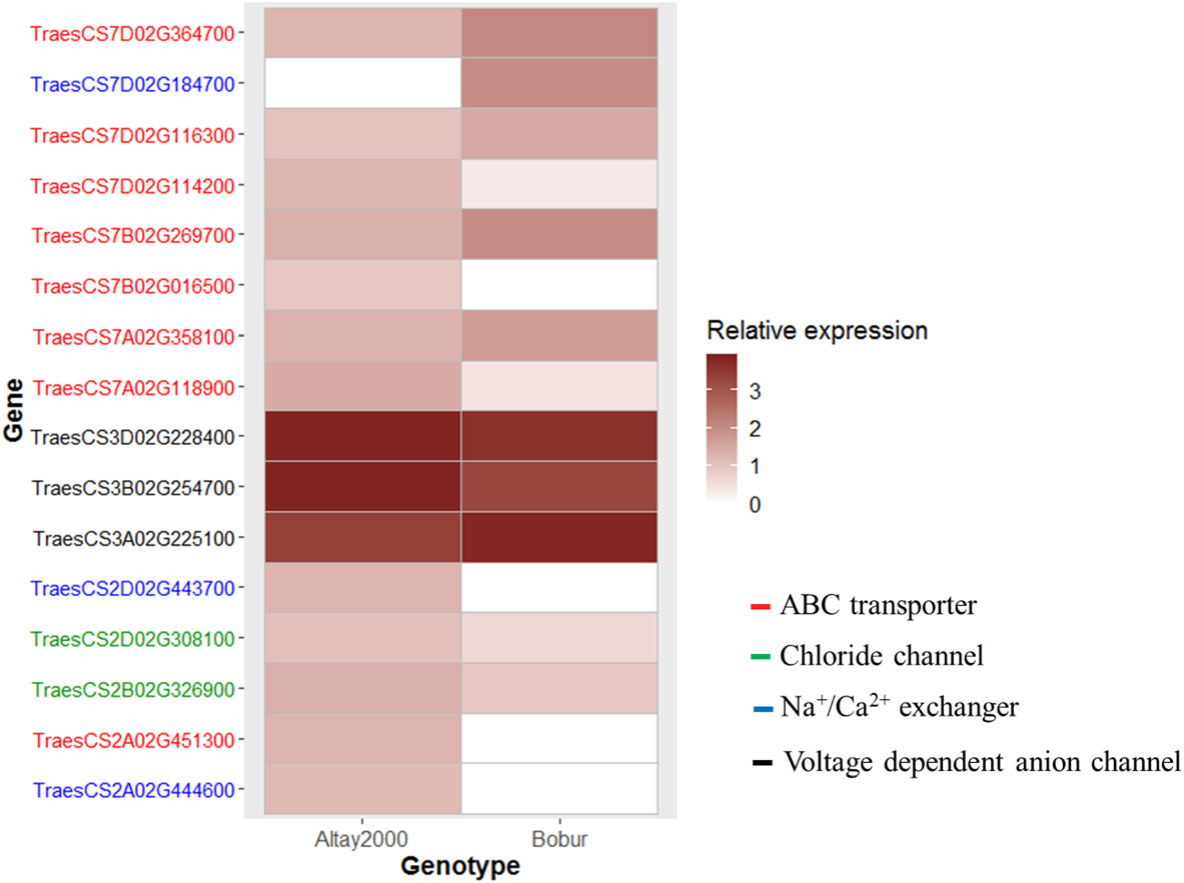
Relative expression (GFOLD value) of transcripts from the transmembrane transport category with a role in ion homeostasis at 24 days after stress

Three GO terms specific for this stress phase were identified, and 11 from 23 categories shared the same stress effect in the two genotypes (Fig. 5c). For instance, chitinase activity and response to oxidative stress were down-regulated in both genotypes while response to water and transmembrane transport were up-regulated (Fig. 5c). Up-regulated transcripts from the transmembrane transport category with potential roles on Na^+^ homeostasis were identified at 24 days ASE (Fig. 7). This analysis revealed a higher number of ABC transporters and Na^+^/Ca^2+^ exchangers expressed in the tolerant genotype but greater relative expression values in the genes expressed in the susceptible genotype. On the other hand, genes involved in translation were down-regulated in the tolerant genotype while genes from the metal ion binding category were up-regulated. The up-regulation of the response to auxin category was observed in the susceptible genotype (Fig. 5c). The Additional file 9 details the over-represented gene categories in the ionic stage, similar to Additional file 6 for the osmotic phase.

### Comparative analysis of osmotic and ionic stress responses

The kinetic of the transcriptomes were compared in the four genotypes to identify genes that changed their expression levels during the osmotic and ionic phases of the salt stress response. Syn86 was the genotype presenting the highest number of salt-responsive genes, from three to five times more genes than the three cultivars. Among all the differentially expressed genes, 86 were stress-responsive in the four genotypes while 50 and 232 transcripts were common in the tolerant and sensitive genotypes, respectively (Fig. 3d).

A total of 20 GO terms were over-represented in both the osmotic and ionic phases (Fig. 5). The translation category was down-regulated in the salt-sensitive Syn86 at the osmotic phase and the tolerant Altay2000 at the ionic phase. The term serine-type endopeptidase inhibitor activity presented opposite relative expression values in the tolerant genotypes of both salt stress phases. These genes were down-regulated in the tolerant genotype and up-regulated in the salt-sensitive one during the osmotic phase. On the contrary, this category showed up-regulation in the salt-tolerant genotype at the ionic stress phase. The response to oxidative stress category was both up- and down-regulated in the contrasting genotypes from the osmotic stress phase, while it was only down-regulated in both genotypes studied during the ionic phase.

### Identification of candidate QTGs

**Fig. 8:**
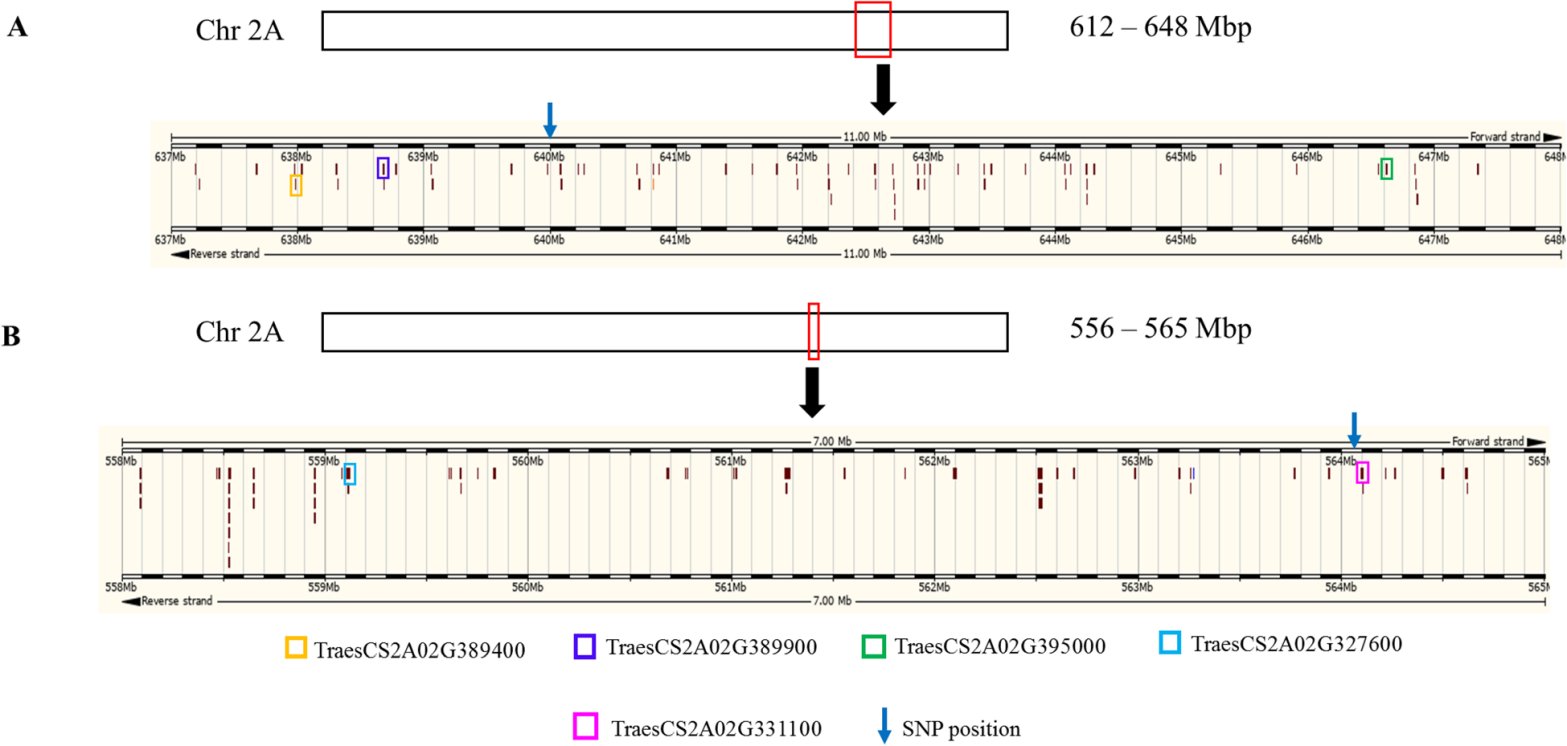
Overview of salt-responsive genes in QTL intervals in chromosome 2A. (A) Marker RAC875_c38018_278 detected by association mapping [28] and (B) marker BS00041707_51 detected by AB-QTL mapping [27]. Salt-responsive genes are marked with colors. The chromosome regions were retrieved from Ensembl Plants release 46 [41]

To unravel candidate QTGs that might contain alleles controlling salt stress-related traits, salt-responsive transcripts within QTL intervals were identified. Syn86 and Zentos are contrasting parents of an advanced backcross-QTL (AB-QTL) study [27], while Altay2000 and Bobur are contrasting genotypes identified in an association panel [28]. Figure 8 and Table 2 present the candidate QTGs from two QTL on the chromosome 2A identified in these studies. A 36 Mbp linkage disequilibrium (LD) block included the single nucleotide polymorphism (SNP) markers RAC875_c38018_278, which was associated with shoot fresh weight after salt stress in the association panel. Three differentially expressed genes were found in this region, one salt-responsive in the sensitive genotype and two in the tolerant one (Table 2, Fig. 8). *TraesCS2A02G395000* showed the strongest stress response as the gene coding an oxoglutarate/iron-dependent dioxygenase was suppressed in the salt-susceptible genotype with an expression value of − 2.4. On the AB-QTL mapping study, a 9 Mbp LD block contained the marker BS00041707_51, which was identified with an effect on kernel weight variation under stress. This region included two up-regulated genes in Syn86 with similar expression levels that coded for an amino acid transporter and a copper amine oxidase. The five candidate QTGs were stress-responsive in other studies (Table 2).

**Table 2:** Differentially expressed genes in LD blocks of markers with effect on salt stress-related traits in the Chr 2A

Marker^**a**^ R^**2**^(%), study	Gene relative expression^**b**^	Annotation^**c**^	Abiotic stress effect^**d**^
RAC875_c38018_278	*TraesCS2A02G389400*: **1.5**	Leucine zipper, homeobox-associated	[42] **↑**, [43] **↑**
	*TraesCS2A02G389900*: **1.0**	Glutamate dehydrogenase	[42] **↑**, [43] **↑**
12.98, [28]	*TraesCS2A02G395000*: *−2.4*	Oxoglutarate/iron-dependent dioxygenase	[42] **↓**
BS00041707_51	*TraesCS2A02G327600*: *2.1*	Copper amine oxidase	[42] **↑**, [43] **↑**
12.5, [27]	*TraesCS2A02G331100*: *1.8*	Amino acid transporter	[42] **↑**

^a^Marker names according to Wang et al. [44]

^b^GFOLD values from tolerant genotypes are bold and from the susceptible are in italics

^c^Based on the Interpro results from the RefSeq v1.0 annotation [45]

^d^Abiotic stress response based on transcriptomics studies of drought and heat [42] and cold [43] deposited in the wheat expression atlas expVIP [46]. The direction of the arrows indicates the stress effect on expression, **↑** when the gene is up-regulated and **↓** when it is down-regulated

### Real-time quantitative PCR analysis

**Fig. 9:**
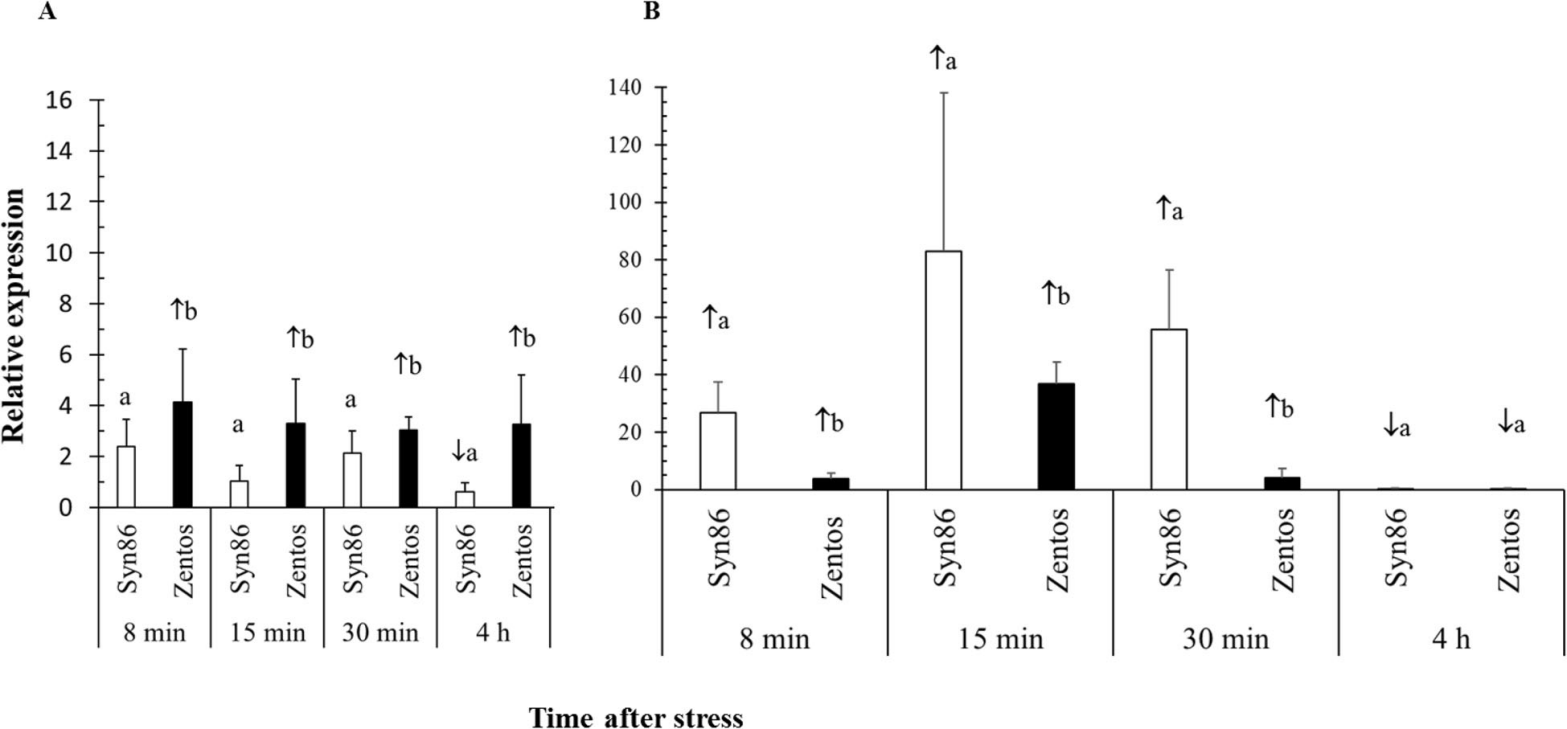
Relative expression values calculated with the ∆∆ Ct method [47]. (A) *TraesCS2D02G173600* expression with *TaEf-1.2* as internal control. (B) *TraesCS5D02G238700* expression with *TaEf-1.1* as internal control. Different letters show significant differences in mean values from the two genotypes (*p* < 0.05). Mean relative expression values > 2.0 or < 1.0 (p < 0.05) indicated up-regulation (**↑**) or down-regulation (**↓**) of genes, respectively

The expression of two genes from the calcium-binding category was analyzed by real-time quantitative PCR (RT-qPCR) to validate the transcript abundance determined with MACE sequencing. Additional file 10 shows the amplification efficiency comparisons of the targets and two reference genes. Based on these results, a different reference gene was selected for each target gene (Fig. 9). The melting curves of the PCR products included in the Additional file 11, revealed single peaks that indicated specific amplification and absence of primer dimers. The expression of *TraesCS2D02G173600* was higher in Zentos than in Syn86 in all the time points studied. This salt-responsive gene was separated by 9 kb from SNP marker Kukri_rep_c72254_186 with an effect on plant biomass under salt stress in the AB-QTL study [27]. Different from the previous gene, *TraesCS5D02G238700* showed higher relative expression values in both genotypes, and in this case, the gene expression in Syn86 was higher than in Zentos (Fig. 9). The relative expression values from the transcriptome and the RT-qPCR analyses were compared in the Additional file 12. The RT-qPCR analysis of *TraesCS5D02G238700* confirmed the greater up-regulation detected in Syn86 in the transcriptomic analysis at 8 and 15 min. In the case of *TraesCS2D02G173600*, the RT-qPCR and transcriptomic relative expression values were less associated.

## Discussion

This study revealed a wide diversity of transcriptional changes resulting from the salt stress application at the osmotic and ionic phases in the genotypes studied. The osmotic stress experiment revealed some rapid transcriptional changes that might be relevant in the early reaction to salt stress to trigger the differential stress acclimation responses of the contrasting genotypes. The initial up-regulation and the posterior down-regulation of photosynthesis-related transcripts in the susceptible genotype were consistent with the observed photosynthesis response. The up-regulation at 8 min of the electron transport in PSII category can be linked to the over-excitation of this system, which leads to an increase in ROS production [14, 48]. The down-regulation of photosynthesis-related genes at 30 min ASE might be a consequence of excessive ROS accumulation that inhibits the repair of photodamaged PSII at both transcriptional and translational levels [49–52]. Nevertheless, the results indicate that plants can recover the expression levels of photosynthesis-related genes as the transcriptional suppression of photosynthesis was not observed at 4 h ASE (Fig. 6a).

The reduced oxidative stress response of Zentos can be attributed to a restrained ROS production, which might stimulate the growth under stressful conditions [52]. Therefore, the reduced photosynthesis inhibition of this genotype can be linked to lower oxidative damage to the photosynthetic apparatus. On the other side, the susceptible genotype revealed the down- and up-regulation of genes implicated in oxidative damage protection with higher relative expression values than the tolerant genotype (Fig. 6b). These results indicate that salt stress exerted a stronger effect on the oxidative damage protection system of Syn86 at the transcriptional level supporting its greater photosynthesis inhibition [52]. Additional studies of ROS contents under stress would be beneficial to link them with the observed transcriptional changes in the contrasting genotypes.

The over-representation of genes coding for calcium-binding proteins at 15 min in Zentos agrees with earlier timing of calcium and ROS signaling proposed for salt-tolerant genotypes [13]. These molecules interact in signaling pathways to regulate salt stress response and trigger systemic responses [13, 14, 17, 53]. The delayed Ca^2+^/ROS signaling observed in Syn86 at 30 min can lead to the activation of the jasmonic acid signaling pathway that will culminate in cell death. Differently, the earlier activation of calcium- and ROS-dependent signaling in the tolerant genotype can induce a constraint on jasmonic acid signaling through the activation of the abscisic acid signaling pathway [13]. In addition to a delayed calcium-binding up-regulation, the salt-driven suppression of calcium-binding genes related to photosynthesis was observed. This result is also related to the photosynthesis inhibition observed in Syn86.

The present study also revealed the differential response of the xyloglucan:xyloglucosyl transferase activity term in the contrasting genotypes. The increased transcription observed in the tolerant genotype might enable plant growth under stress and might be beneficial for cell wall strengthening, the prevention of excessive water loss and the maintenance of turgor pressure due to the biosynthesis of xyloglucan in the cell wall [54, 55]. The synergy and the specific timing of the described transcriptional events might be crucial for the differential stress response of the genotypes studied at this early stress phase. The rapid signaling events occurring in Zentos might be linked to the activation of beneficial mechanisms for stress adaptation and greater photosynthesis stability.

The similar stress response of some GO terms observed in the contrasting genotypes at the ionic stress phase suggests that some earlier transcriptional responses might present more substantial differences and might significantly impact the contrasting acclimation response of the genotypes to long-term salt stress [15]. Nevertheless, it is also possible that when similar categories are salt-responsive in both genotypes, the difference might lie in the specific genes and their levels of expression to affect the differential stress response. For instance, the transmembrane transport category was up-regulated in both genotypes. This category contained ABC transporters and Na^+^/Ca^2+^ exchangers exclusively expressed in the tolerant genotype that can be relevant for Na^+^ exclusion mechanisms [56–58]. Further experiments are needed to confirm the link of the stress-induced expression of these genes and the reduced Na^+^ accumulation discovered in the tolerant genotype at the ionic phase [59].

According to the results, the over-represented GO categories from the salt-responsive genes and their suppression or over-expression were in line with the physiological measurements performed in the contrasting genotypes at both stress phases. Most of the GO terms over-represented in the ionic phase were also found in the osmotic phase, which indicated a set of common transcriptional responses at these stress phases. There is an opposite regulation of the translation and serine-type endopeptidase inhibitor categories across both stress phases, suggesting that the control of these mechanisms is stress-stage-specific. The accumulation of aberrant proteins in cells can result from stress-related ROS damage, which can lead to the transient suppression of the de novo synthesis of proteins and the intracellular protein degradation by proteases [60–62].

RT-qPCR demonstrates the accuracy of transcriptomic results due to the multiple sources of biases that can occur in the procedures of sample preparation, RNA extraction and sequencing along with the complex pipeline required for the analysis of the libraries [63–65]. RT-qPCR confirmed TraesCS5D02G238700 up-regulation at the osmotic phase, while the up-regulation of *TraesCS1B02G144500* (*ZIP7*) at the ionic phase was corroborated in the study by Oyiga et al. [28]. The poor concordance of the expression of *TraesCS2D02G173600* in the two platforms might result from the high frequency of multiple aligned reads in some genomic regions that can lead to expression quantification biases in the transcriptomic analyses [66].

From the three salt-responsive genes observed in the QTL interval from the association mapping analysis, the oxoglutarate/iron-dependent dioxygenase showed the strongest down-regulation in the susceptible genotype. This gene superfamily might be involved in the biosynthesis of several specialized secondary metabolites responsive to biotic and abiotic stresses [67, 68]. Therefore, this gene is a strong candidate that can be prioritized for further validation analyses.

The AB-QTL mapping interval contained two salt-responsive genes, including a copper amine oxidase and an amino acid transporter with similar magnitudes of relative expression. The up-regulation of both genes in the sensitive genotype can be linked to the positive phenotypic effect of the allele from Syn86 in the variation of kernel weight under salt stress [27]. Studies in *Arabidopsis* have shown the involvement of copper amine oxidases in the biosynthesis of nitric oxide, which is a signaling molecule that participates in adaptive responses to biotic or abiotic stresses [69–71]. On the other hand, amino acid transporters up-regulated by salt stress can be involved in the transport of amino acids, such as proline, which accumulates under stress to act as an osmolyte for osmotic adjustment [72, 73]. The differential expression of the genes in the interval may contribute concomitantly to the phenotypic variation [30]. The RT-qPCR validated *TraesCS2D02G173600* is another example of a candidate gene within a QTL from the AB-QTL study in D subgenome [27]. In this case, the delimitation of an LD interval was not possible because of the low SNP-marker density resulting from the reduced genetic variability of this subgenome [27, 74, 75].

Due to the high complexity of the bread wheat genome and the low resolution of mapping studies, it is mandatory to implement strategies to pinpoint potential functional candidates in QTL intervals to get insights into the mechanistic basis of complex traits. The approach developed in this study highlights the usefulness of the recent fully annotated and highly contiguous chromosome-level reference genome sequence assembly to facilitate the integration of genomic and transcriptomic resources to resolve QTL in bread wheat [5, 76]. This strategy can be more robust when expression data of other tissues under salt stress and additional time points can also be included. Further steps to confirm the causality of the selected candidate genes on the traits of interest are the identification of polymorphisms in coding and promoter regions, and the combination of a higher resolution mapping approach with functional studies.

Besides uncovering the dynamic transcriptome during the salt stress response and uncovering QTGs in QTL intervals, the MACE-derived sequence analysis conferred evidence of two types of novel transcribed regions in bread wheat. Firstly, novel transcripts involved in the salt stress response were identified. These transcripts might enrich the wheat variable pangenome that represents 39% of the pangenome according to the analysis of the whole genome of 18 cultivars [77]. Secondly, the detection of reads beyond the predicted 3′-ends of gene models indicates prolonged transcription and can contribute to the improvement of the RefSeq v1.1 annotation [5]. The discovery of these reads suggests that some current gene model predictions were based on transcripts with incomplete read coverage in the 3′-ends. The genes identified with extended transcription can be included in computational prediction approaches to refine gene structures [39, 78]. The consideration of these additional reads was relevant to perform a better quantification of expression levels because with the current reference genome annotation these reads were ignored. The RT-qPCR validation of both novel transcripts and 3′-ends is necessary to confirm the transcription of these regions.

## Conclusions

This presented study highlights key gene categories affected at the transcription level during the osmotic and ionic phases of the salt stress response. We inferred that cell wall synthesis and calcium-binding genes activated early in the tolerant genotype at the osmotic phase might be relevant in mechanisms to trigger the greater photosynthesis stability and the overall increased salt stress acclimation from this genotype. The specific up-regulation of some ABC transporters and Na^+^/Ca^2+^ exchangers in the tolerant genotype at the ionic stage indicates their involvement in sodium exclusion mechanisms. We expect that our results will encourage the wheat research community to perform functional analysis of some prioritized genes within QTL intervals to follow a step of development of allele-specific primers to use in marker-assisted selection approaches. These results will lead to a better QTL dissection to finally shed light on novel genes controlling regulatory pathways for salt stress-related traits that can be further utilized in wheat breeding programs.

## Methods

### Contrasting genotypes from the mapping populations and tissue sampling

The elite German winter wheat cultivar Zentos (salt-tolerant) and the synthetic genotype Syn86 (salt-susceptible) were the contrasting parents from an AB-QTL study used to analyze the foliar transcriptome during the osmotic stress response [27, 79]. Seeds of Zentos were provided by Syngenta Seeds GmbH (Bad Salzuflen, Germany), while Syn86 seeds were produced and supplied by Lange and Jochemsen [80]. The Turkish cultivar Altay2000 (salt-tolerant) and the Uzbek cultivar Bobur (salt-susceptible) were the winter wheat genotypes identified in an earlier study as contrasting for shoot accumulation of K^+^ and Na^+^ at 24 days ASE among other stress-related traits [59]. These genotypes included in a panel used in association mapping studies [28, 29], were selected to analyze the leave transcriptome during the ionic stress response. The seeds for the study were delivered by the International Winter Wheat Improvement Program, as described in Oyiga et al. [28, 29, 59].

The contrasting genotypes were grown on hydroponic systems in a growth chamber with 20 ± 2 °C, 50 ± 5% humidity, 12 h photoperiod with four lamps having a light intensity of 200 μmol m^− 2^ s^− 1^ and a salt stress treatment of 100 mM NaCl. The detailed procedures of the hydroponic systems are found in Dadshani et al. [81] and Oyiga et al. [59] for osmotic and ionic phase experiments, respectively. Briefly, for osmotic stress sampling, the seeds were germinated in Petri boxes (29.0 × 22.5 × 3.0 cm; Licefa GmbH & Co. KG, Bad Salzuflen, Germany) with filter paper (C160; Munktell & Filtrak GmbH; Bärenstein, Germany) and distilled water. After eight days, 54 healthy seedlings were transferred to sponges inside holes of styrodur panels (Styrodur 3035 CS; BASF, Ludwigshafen, Germany) placed over dark polypropylene boxes filled with 170 L of nutrient solution continually aerated by four air diffusers (Eheim 4,002,650, Eheim GmbH & Co. KG, Deizisau, Germany). Seedlings of a uniform size adapted for ten days to hydroponics conditions were used for sampling leaves in time points based on the photosynthesis response analyzed by Dadshani [27]. A differential stress response in these genotypes was revealed at this stress phase by measuring the time course of the photosynthesis rate using a gas exchange system (LI-6400XT; LI-COR Environmental, Lincoln, NE, USA). This analysis was performed in a hydroponic system using five 50 days old plants that were then transferred into the NaCl solution. Data were recorded from the third fully expanded leaf in 30 s intervals until 45 min ASE. This study allowed the identification of the turning points of the photosynthesis rate. Turning points referred to the time points with maximum variation response revealed by the change of direction from the curve slope, as shown in Fig. 2 [82]. Therefore, osmotic stress conditions were sampled in the photosynthesis turning time points identified at 8, 15 and 30 min (Fig. 2), and also at 4 h ASE, whereas control conditions were sampled at 0, 30 min and 4 h in plants grown in hydroponic boxes without NaCl.

The hydroponic system used at the ionic phase considered the use of dark polypropylene boxes of 170 L capacity filled with 156 PVC tubes (4.5 cm diameter × 45 cm depth) and 164 L of nutrient solution. In this case, the seeds were germinated in-situ in tubes containing Aquagran filter quartz 2–3.15 mm (Euroquarz GmbH, Dorsten, Germany). Three days after planting, salt was applied gradually during 3 days until the final concentration was reached [59]. Samples for the ionic stress conditions were collected at 11 days and 24 days in both salt-stressed and control plants. Equal amounts of leaf tissue from four plants were harvested and homogenized in liquid nitrogen to constitute one RNA pool for each control and stress time point.

### MACE reads processing and mapping to the reference genome

The total RNA of pooled samples was isolated using the method developed by Chang et al. [83], and 5 μg were used for cDNA synthesis. The MACE library construction protocol was performed as described in Zawada et al. [84] with an Illumina NextSeq 500 system that sequenced biotinylated 3′-ends fragments from 16 to 200 bp. The Cutadapt tool was used to remove adapters from reads [85]. These procedures were carried out at GenXPro GmbH (Frankfurt, Germany), where 14 and 8 libraries from the osmotic and ionic stress experiments were generated, respectively. The quality control of the libraries was carried out using FastQC [86], and the short reads with less than 35 bp were removed with Trimmomatic [87]. The retained reads were aligned to the reference genome assembly version “RefSeq v1.0” [5] using Tophat [88]. Assemblies of novel transcripts were produced with the prediction tool of Cufflinks [89, 90]. The markdup tool from SAMtools was employed to generate deduplicated alignment files and estimate the amount of read duplication [91].

To better estimate gene expression levels and thus to contribute to gene model improvement, a new annotation file was created to count reads beyond the predicted 3′-ends of HC and LC gene models [5]. Thus, the gene models from the RefSeq v1.1 genome annotation [5] were extended by 40% downstream of the predicted 3′-end in the case of intergenic regions greater than 1000 bp but smaller than three times the gene size. When the intergenic distance was larger, the elongated target sequence corresponded to the size of the gene. Then, the stranded option from the featureCounts tool of the Subread package [92, 93] was used to count the unique mapped reads assigned to the elongated HC and LC gene models, and novel predicted transcripts.

The read count data were normalized to counts per million. A transcriptomic background was defined in each genotype to reduce the number of low expressed transcripts that might cause sampling noise [94, 95]. Therefore, an average normalized value of 2.5 counts per million across libraries from the same genotype was selected as a threshold to filter out less than 5% of reads in each library (see Additional file 1).

### Identification of salt-responsive genes and gene ontology enrichment analysis

After filtering transcripts with few reads, salt-responsive genes were identified using the raw count data of fragments as input in the GFOLD software [40]. The GFOLD value is a reliable estimator of the relative gene expression developed for the analysis of pooled experiments [40]. Density plots with log_10_ normalized expression values were used to compare their distributions. Overlapping expression distributions indicate appropriate homogeneity of the sequencing depth and that count normalization is suitable to compare the expression levels of the different libraries [96]. The sample at 0 min was used as a control for both 8 and 15 min ASE, assuming that few physiological changes occur in this short time under normal conditions. A high absolute GFOLD value indicated greater up- or down-regulation of the genes. Genes with GFOLD values > 1 or < − 1 when c = 0.01 were considered for further analyses as they represent relevant changes in expression levels under stress conditions. The defined c value is a parameter that indicated that in 99% of the cases, the fold change of a gene is above the absolute GFOLD (0.01) value calculated for this gene [40].

The GO enrichment tool from the STEM software was implemented to distinguish the categories of genes over-represented by time point in the contrasting genotypes [97, 98]. Only the gene categories from the transcriptomic background of each genotype were retained in the analysis [99]. A Bonferroni multiple hypothesis correction test was employed, thus GO terms with a corrected *p*-value < 0.001 were considered as over-represented. The STEM algorithm was used to cluster expression profiles during the osmotic phase, and the over-represented GO categories in the clusters were defined [97, 98]. Key categories over-represented during the osmotic phase were selected to create graphs of the corresponding genes’ time-course expression. A LOESS model was fitted to represent the expression tendency of the clusters of genes. The expression levels of transcripts from the transmembrane transport category related to ion homeostasis were compared in the contrasting genotypes at the ionic phase.

### Identification of QTGs

QTL intervals were delimited through the identification of SNPs in strong LD (R^2^ ≥ 0.8) with markers detected with a significant effect on trait variation [100]. QTGs were identified by localizing salt-responsive genes detected with the transcriptomic analysis within the LD blocks spanning the QTL [27, 28]. The positions of the LD blocks in the reference genome sequence RefSeq v1.0 were established according to the IWGSC RefSeq v1.0 BLAST results of the SNPs-flanking sequences [45]. The wheat RNA-seq atlas expVIP was used to compare the expression of the QTGs with the expression determined in other abiotic stress experiments in the species [46].

### Real-time quantitative PCR analysis

A new hydroponic experiment with Syn86 and Zentos was carried out following the procedures described previously for these genotypes and using a salt treatment of 150 mM NaCl. Both stress and control conditions were sampled at the time points determined for the osmotic phase. Three seedlings from each condition were collected and frozen in liquid nitrogen. Leaves were manually grounded, and 200 mg of tissue from each plant was used for total RNA isolation with the RNeasy plant mini kit (Qiagen, Hilden, Germany). The cDNA synthesis was performed with the First Strand cDNA Synthesis Kit (Thermo Scientific, Waltham, MA, USA) using 5 μg of total RNA from each sample. The SDS-7500 Sequence Detection System (Applied Biosystems, Waltham, MA, USA) was used for RT-qPCR with cycling conditions of 95 °C/7 min followed by 40 cycles at 95 °C/10 s, 60 °C/30 s, 72 °C/30 s, and 82 °C/30 s (fluorescence acquisition). Subgenome-specific primers for the salt-responsive genes *TraesCS2D02G173600* and *TraesCS5D02G238700* were designed using the web-based tool GSP [101] and are available in the Additional file 10. The amplification efficiencies of the internal control primers *TaEf-1.1 and TaEf-1.2* [28, 29] and each target gene were compared. The RT-qPCR reaction of 20 μl consisted on 0.25 μM of each primer, 10.12 μl of DyNamo Color Flash SYBR Green 2X-master mix with ROX (Thermo Scientific, Waltham, MA, USA) and 2.5 μl of 1:20 diluted cDNA template. One cycle of 95 °C for 10 s, 60 °C for 30 s and 95 °C for 15 s was applied to PCR products for melting curve analysis.

The average Ct values of three technical replicates were calculated and used as input for quantifying the relative expression of the selected genes using the ∆∆ Ct method [47]. A one-sample single-tailed t-test (*p* < 0.05) was implemented in the 2^−∆∆*Ct*^ values of each genotype per time point to assess whether the mean was > 2.0 or < 1.0 to define when the transcripts were up-regulated or down-regulated upon stress, respectively. A two-sample two-tailed t-test (p < 0.05) was used to compare the 2^−∆∆*Ct*^ values from the two genotypes to determine if the mean relative expression values were significantly different in each time point. The mean ∆∆ Ct values and the GFOLD values obtained from the transcriptomic analysis were compared.

## Supplementary information

**Additional file 1: Table S1.** Overview of reads processing and reference genome mapping from all libraries.

**Additional file 2: Table S2.** Genes with extended 3′-end and position in the reference genome of the clusters of additional reads scored.

**Additional file 3: Fig. S1.** Density plots with the log_10_ normalized expression values of the libraries from the four genotypes studied.

**Additional file 4: Table S3.** Reference genome coordinates from the novel salt-responsive transcripts identified in the four genotypes.

**Additional file 5: Table S4.** GFOLD values of the salt-responsive genes identified in the time points studied in the four genotypes.

**Additional file 6: Table S5.** Details of over-represented gene categories during the osmotic phase.

**Additional file 7: Fig. S2.** Clusters of expression profiles of Syn86 and Zentos.

**Additional file 8: Table S6.** Gene ontology terms over-represented in the gene clusters from Syn86 and Zentos.

**Additional file 9: Table S7.** Details of over-represented gene categories during the ionic phase.

**Additional file 10: Table S8.** RT-qPCR primers of target genes and amplification efficiency assessment.

**Additional file 11: Fig. S3.** Melting curves of some PCR products derived from the amplification of reference and target genes.

**Additional file 12: Fig. S4.** Comparison of GFOLD values and ∆∆ Ct values of studied genes.

## Data Availability

The main data supporting the results of this article are included within the article and the provided additional files. The files with aligned reads of each library were deposited to the Sequence Read Archive (SRA) of the National Center for Biotechnology Information in the BioProject with accession number PRJNA549411.

## Abbreviations

AB-QTL: advanced backcross-QTL
ASE: after stress exposure
GA: genes assigned to the category
GE: genes expected in the category
GFOLD: generalized fold change
GO: gene ontology
HC: high confidence
IWGSC: International Wheat Genome Sequencing Consortium
LC: low confidence
LD: linkage disequilibrium
LOESS: locally estimated scatterplot smoothing
MACE: Massive Analysis of cDNA 3′-ends
PSII: photosystem II
QTG: quantitative trait gene
QTL: quantitative trait loci
ROS: reactive oxygen species
RT-qPCR: real-time quantitative PCR
