# Supplementary material for: Transcriptome profiling at osmotic and ionic phases of salt stress response in bread wheat uncovers trait-specific candidate genes
Source: BMC Plant Biol. 2020 Sep 16;20:428. doi: 10.1186/s12870-020-02616-9 (PMC7493341; doi:10.1186/s12870-020-02616-9)
Supplement: Supplementary file 3 — Additional file 3: Fig. S1. Density plots with the log10 normalized expression values of the libraries from the four genotypes studied. [file 12870_2020_2616_MOESM3_ESM.pdf]

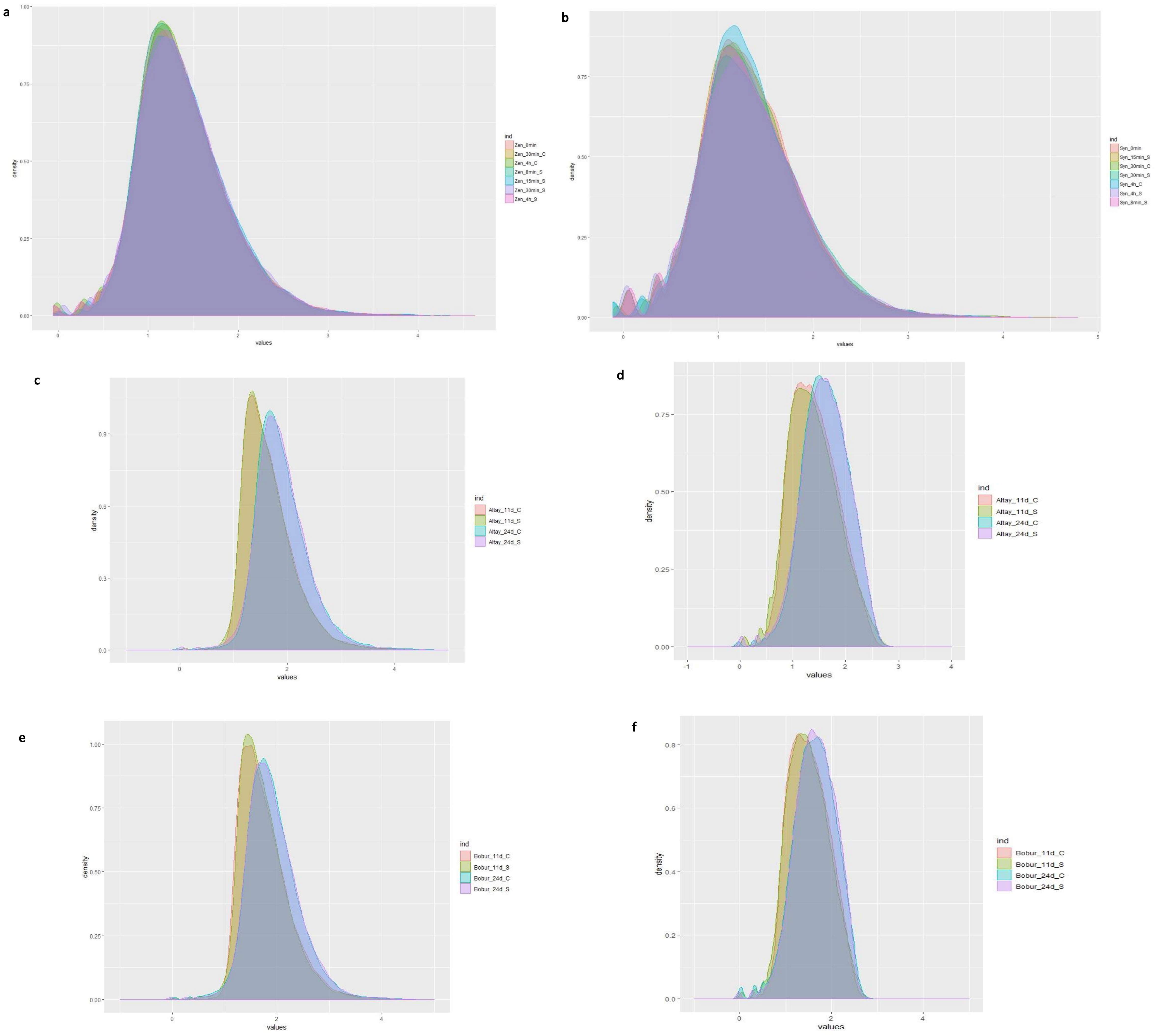

Figure S1. Density plots with the $\log_{10}$ normalized expression values of the libraries from the four genotypes studied. a) Zentos, b) Syn86, c) Altay2000 without deduplication, d) Altay2000 deduplicated, e) Bobur without deduplication and f) Bobur deduplicated. C= Control library, S= Stress library.
