# Supplementary material for: Transcriptome profiling at osmotic and ionic phases of salt stress response in bread wheat uncovers trait-specific candidate genes
Source: BMC Plant Biol. 2020 Sep 16;20:428. doi: 10.1186/s12870-020-02616-9 (PMC7493341; doi:10.1186/s12870-020-02616-9)
Supplement: Supplementary file 7 — Additional file 7: Fig. S2. Clusters of expression profiles of Syn86 and Zentos. [file 12870_2020_2616_MOESM7_ESM.pdf]

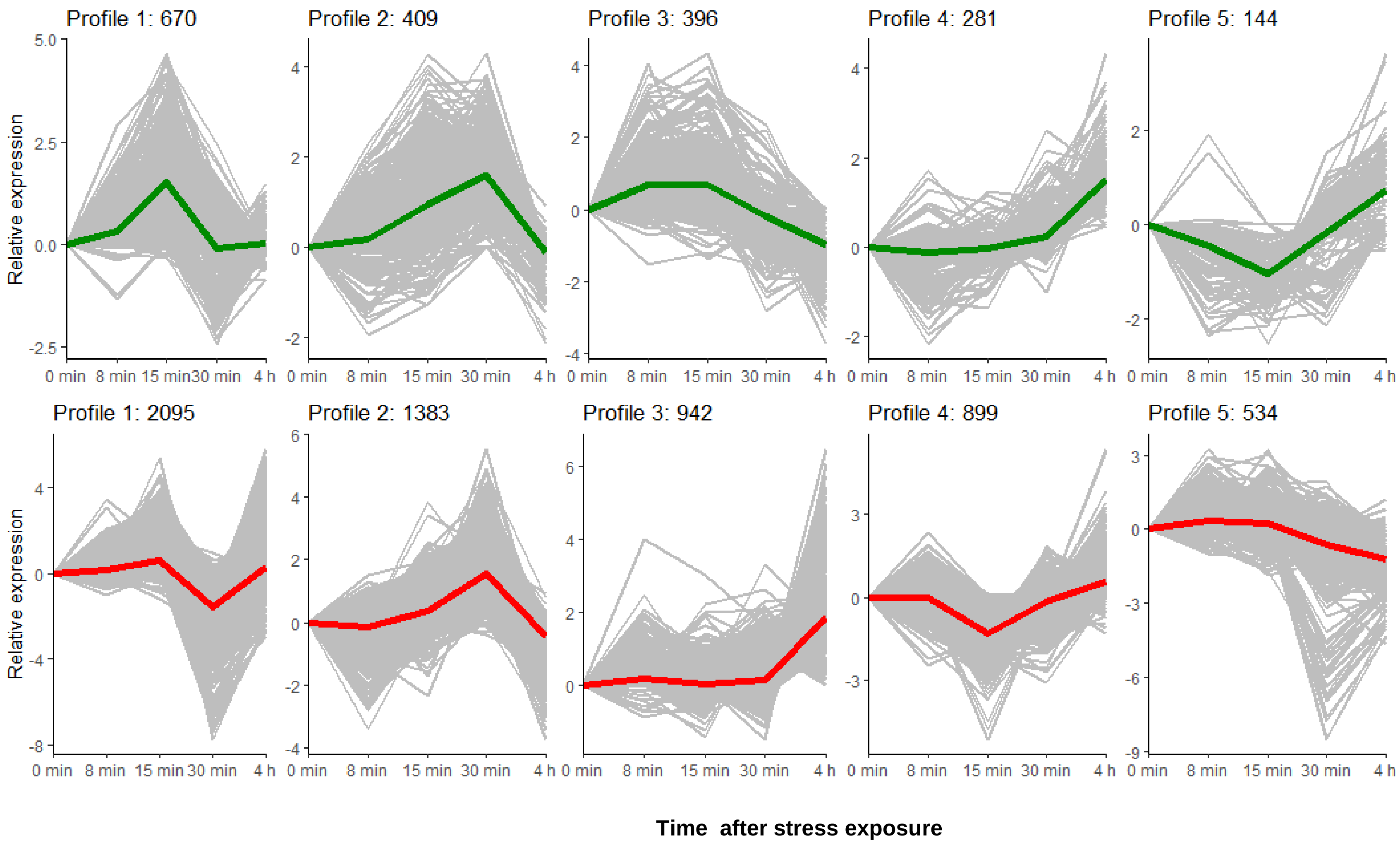

Fig. S2. Clusters of expression profiles of Syn86 (red) and Zentos (green) with the number of genes indicated in each one. In each expression profile frame, the gray lines show the time course expression pattern of each gene and the red or green lines are LOESS (locally estimated scatterplot smoothing) curves that represents the expression tendency of the clusters of genes
