# Supplementary material for: Transcriptome profiling at osmotic and ionic phases of salt stress response in bread wheat uncovers trait-specific candidate genes
Source: BMC Plant Biol. 2020 Sep 16;20:428. doi: 10.1186/s12870-020-02616-9 (PMC7493341; doi:10.1186/s12870-020-02616-9)
Supplement: Supplementary file 8 — Additional file 8: Table S6. Gene ontology terms over-represented in the gene clusters from Syn86 and Zentos. [file 12870_2020_2616_MOESM8_ESM.pdf]

Table S5. GO terms over-represented in the gene clusters from Syn86 and Zentos

a. Zentos

Profile 1

Category ID	Category Name	Genes Category	Genes Assigned	Corrected p-value
GO:0003700	transcription factor activity, sequence-specific DNA binding	492	33	0.00000025
GO:0006869	lipid transport	28	9	0.0000012
GO:0004672	protein kinase activity	1404	57	0.000027
GO:0006468	protein phosphorylation	1407	57	0.000029
GO:0009415	response to water	12	6	0.000033
GO:0005509	calcium ion binding	432	26	0.00017
GO:0006355	regulation of transcription, DNA-templated	881	39	0.00057

Profile 2

Category ID	Category Name	Genes Category	Genes Assigned	Corrected p-value
GO:0043565	sequence-specific DNA binding	318	39	1.5^{-25}
GO:0003700	transcription factor activity, sequence-specific DNA binding	492	46	2.7^{-25}
GO:0006355	regulation of transcription, DNA-templated	881	56	9^{-23}
GO:0016702	oxidoreductase activity, acting on single donors with incorporation of two atoms of oxygen	38	8	0.0000088
GO:0046872	metal ion binding	512	22	0.00011
GO:0004601	peroxidase activity	118	10	0.00097

Profile 3

Category ID	Category Name	Genes Category	Genes Assigned	Corrected p-value
GO:0048046	apoplast	33	14	1.8^{-16}
GO:0016762	xyloglucan:xyloglucosyl transferase activity	30	13	2.5^{-15}

GO:0006073	cellular glucan metabolic process	30	13	2.5^{-15}
GO:0004553	hydrolase activity, hydrolyzing O-glycosyl compounds	272	27	4.7^{-15}
GO:0005618	cell wall	34	13	1.9^{-14}
GO:0010411	xyloglucan metabolic process	26	10	9.6^{-11}
GO:0005975	carbohydrate metabolic process	491	28	1.4^{-9}
GO:0042546	cell wall biogenesis	34	10	2.2^{-9}
GO:0003700	transcription factor activity, sequence-specific DNA binding	492	23	0.0000067
GO:0003677	DNA binding	1152	34	0.00018
GO:0006355	regulation of transcription, DNA-templated	881	28	0.00055

Profile 4

Category ID	Category Name	Genes Category	Genes Assigned	Corrected p-value
GO:0050832	defense response to fungus	11	8	1.9^{-12}
GO:0042742	defense response to bacterium	11	8	1.9^{-12}
GO:0004332	fructose-bisphosphate aldolase activity	19	6	0.0000051
GO:0016998	cell wall macromolecule catabolic process	33	7	0.0000055
GO:0006032	chitin catabolic process	33	7	0.0000055
GO:0004568	chitinase activity	33	7	0.0000055
GO:0016705	oxidoreductase activity, acting on paired donors, with incorporation or reduction of molecular oxygen	251	14	0.000012
GO:0005975	carbohydrate metabolic process	491	19	0.000012
GO:0055114	oxidation-reduction process	1551	35	0.000016
GO:0020037	heme binding	386	16	0.000069
GO:0008061	chitin binding	18	5	0.00021
GO:0005506	iron ion binding	317	14	0.00022

Profile 5

Category ID	Category Name	Genes Category	Genes Assigned	Corrected p-value
GO:0005576	extracellular region	51	10	7.7^{-12}
GO:0004867	serine-type endopeptidase inhibitor activity	23	6	0.00000034

b. Syn86

Profile 1

Category ID	Category Name	Genes Category	Genes Assigned	Corrected p-value
GO:0015979	photosynthesis	136	82	5.5^{-62}
GO:0009523	photosystem II	81	44	1^{-29}
GO:0009522	photosystem I	33	27	4.4^{-25}
GO:0055114	oxidation-reduction process	1652	205	1.8^{-21}
GO:0009654	photosystem II oxygen evolving complex	64	31	1.7^{-18}
GO:0009538	photosystem I reaction center	16	15	5.3^{-15}
GO:0019898	extrinsic component of membrane	52	25	1.7^{-14}
GO:0006869	lipid transport	25	15	6.5^{-10}
GO:0048046	apoplast	39	17	0.000000018
GO:0016655	oxidoreductase activity, acting on NAD(P)H, quinone or similar compound as acceptor	12	10	0.00000003
GO:0016747	transferase activity, transferring acyl groups other than amino-acyl groups	154	33	0.000000087
GO:0015995	chlorophyll biosynthetic process	20	12	0.00000014
GO:0005975	carbohydrate metabolic process	513	69	0.00000016
GO:0016760	cellulose synthase (UDP-forming) activity	46	17	0.00000041
GO:0008152	metabolic process	1043	112	0.00000071
GO:0016702	oxidoreductase activity, acting on single donors with incorporation of two atoms of oxygen	48	17	0.00000088
GO:0016762	xyloglucan:xyloglucosyl transferase activity	37	15	0.00000095

GO:0006073	cellular glucan metabolic process	37	15	0.00000095
GO:0005840	ribosome	606	74	0.0000029
GO:0016491	oxidoreductase activity	957	103	0.000003
GO:0005618	cell wall	41	15	0.0000051
GO:0020037	heme binding	435	58	0.0000063
GO:0030244	cellulose biosynthetic process	56	17	0.000013
GO:0006412	translation	630	74	0.000015
GO:0010411	xyloglucan metabolic process	33	13	0.000019
GO:0005506	iron ion binding	374	50	0.000062
GO:0003735	structural constituent of ribosome	630	71	0.00015
GO:0008289	lipid binding	74	18	0.00021
GO:0008171	O-methyltransferase activity	35	12	0.0004
GO:0042546	cell wall biogenesis	42	13	0.00051
GO:0010333	terpene synthase activity	31	11	0.00083
GO:0016020	membrane	1269	117	0.001
GO:0015986	ATP synthesis coupled proton transport	45	13	0.001

Profile 2

**Category ID**	**Category Name**	**Genes Category**	**Genes Assigned**	**Corrected p-value**
GO:0003735	structural constituent of ribosome	630	170	1^{-90}
GO:0005840	ribosome	606	165	1.4^{-88}
GO:0006412	translation	630	166	1.1^{-86}
GO:0005622	intracellular	709	128	9.1^{-46}
GO:0016998	cell wall macromolecule catabolic process	38	19	2.6^{-14}
GO:0006032	chitin catabolic process	38	19	2.6^{-14}
GO:0004568	chitinase activity	38	19	2.6^{-14}
GO:0020037	heme binding	435	58	3.5^{-13}
GO:0015935	small ribosomal subunit	51	18	3.2^{-10}
GO:0003700	transcription factor activity, sequence-specific DNA binding	536	58	3.1^{-9}

GO:0015934	large ribosomal subunit	48	16	0.000000018
GO:0043565	sequence-specific DNA binding	346	43	0.000000024
GO:0006414	translational elongation	59	17	0.000000059
GO:0004672	protein kinase activity	1471	107	0.00000047
GO:0006468	protein phosphorylation	1473	107	0.00000051
GO:0008061	chitin binding	19	10	0.00000052
GO:0006979	response to oxidative stress	137	24	0.00000066
GO:0004867	serine-type endopeptidase inhibitor activity	44	14	0.00000067
GO:0009611	response to wounding	20	10	0.000001
GO:0004601	peroxidase activity	124	22	0.0000026
GO:0005975	carbohydrate metabolic process	513	50	0.0000035
GO:0016705	oxidoreductase activity, acting on single donors with incorporation of two atoms of oxygen	295	33	0.000065
GO:0003746	translation elongation factor activity	55	13	0.00014
GO:0010333	terpene synthase activity	31	10	0.00016
GO:0016758	transferase activity, transferring hexosyl groups	148	21	0.00032
GO:0004553	hydrolase activity, hydrolyzing O-glycosyl compounds	286	31	0.00033
GO:0006952	defense response	34	10	0.00043
GO:0048544	recognition of pollen	54	12	0.00088

Profile 3

Category ID	Category Name	Genes Category	Genes Assigned	Corrected p-value
GO:0008152	metabolic process	1043	78	2.7^{-13}
GO:0003824	catalytic activity	1463	91	5.8^{-11}
GO:0009415	response to water	19	9	0.00000046
GO:0004867	serine-type endopeptidase inhibitor activity	44	12	0.0000011
GO:0055114	oxidation-reduction process	1652	87	0.0000011
GO:0004350	glutamate-5-semialdehyde dehydrogenase activity	5	5	0.000013
GO:0016491	oxidoreductase activity	957	57	0.000016

GO:0043169	cation binding	97	15	0.000042
GO:0006950	response to stress	143	17	0.00028
GO:0030170	pyridoxal phosphate binding	141	16	0.001

Profile 4

Category ID	Category Name	Genes Category	Genes Assigned	Corrected p-value
GO:0055085	transmembrane transport	669	52	1.2^{-9}
GO:0004014	adenosylmethionine decarboxylase activity	5	5	0.00001
GO:0006597	spermine biosynthetic process	5	5	0.00001
GO:0006814	sodium ion transport	25	8	0.00012
GO:0008295	spermidine biosynthetic process	8	5	0.00054
GO:0006355	regulation of transcription, DNA-templated	919	49	0.001

Profile 5

Category ID	Category Name	Genes Category	Genes Assigned	Corrected p-value
GO:0045735	nutrient reservoir activity	35	17	2.8^{-19}
GO:0030145	manganese ion binding	46	17	9.4^{-17}
GO:0000786	nucleosome	121	17	4.2^{-9}
GO:0003700	transcription factor activity, sequence-specific DNA binding	536	31	0.00000024
GO:0003677	DNA binding	1139	47	0.0000006
GO:0006334	nucleosome assembly	64	11	0.0000029
GO:0006355	regulation of transcription, DNA-templated	919	36	0.00023
