# Supplementary material for: Transcriptome profiling at osmotic and ionic phases of salt stress response in bread wheat uncovers trait-specific candidate genes
Source: BMC Plant Biol. 2020 Sep 16;20:428. doi: 10.1186/s12870-020-02616-9 (PMC7493341; doi:10.1186/s12870-020-02616-9)
Supplement: Supplementary file 10 — Additional file 10: Table S8. RT-qPCR primers of target genes and amplification efficiency assessment. [file 12870_2020_2616_MOESM10_ESM.pdf]

Table S6. RT-qPCR primers of target genes and amplification efficiency assessment with the reference genes *Ef1.1* and *Ef1.2* (Oyiga *et al*., 2018, 2019).

Gene	Forward 5'-3'	Reverse 5'-3'	Amplicon size (bp)	Efficiency (slope)	
				Ef1.1	***Ef1.2***
TraesCS2D02G173600	TCTGCTGTGCTATGCTCGAC	TCCCGTTCTGTCCGAATTCTA	250	-0.1038	0.0432
TraesCS5D02G238700	TCGTTTGTTCGTTTGTTCGTTTG	GTATAAAGAATAGGATTGGATACA	110	-0.043	-0.167
