# Supplementary material for: Transcriptome profiling at osmotic and ionic phases of salt stress response in bread wheat uncovers trait-specific candidate genes
Source: BMC Plant Biol. 2020 Sep 16;20:428. doi: 10.1186/s12870-020-02616-9 (PMC7493341; doi:10.1186/s12870-020-02616-9)

Figure S3. Melting curves of some PCR products derived from the amplification of reference and target genes.

a) TraesCS2D02G173600

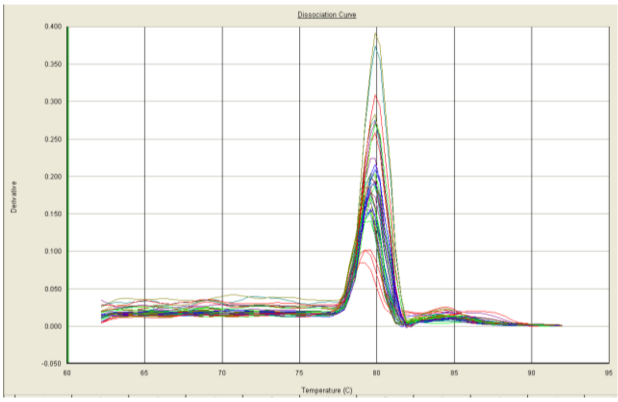

b) TraesCS5D02G238700

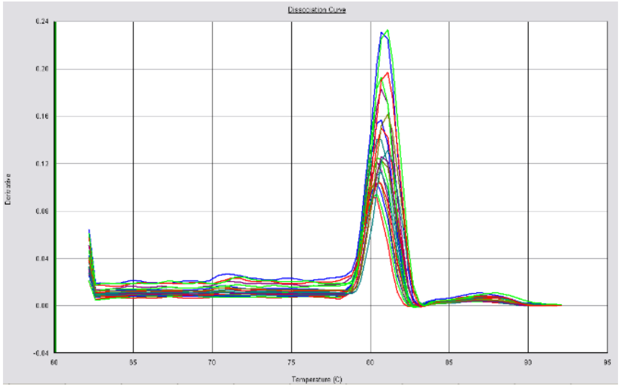

c) Ef1.1

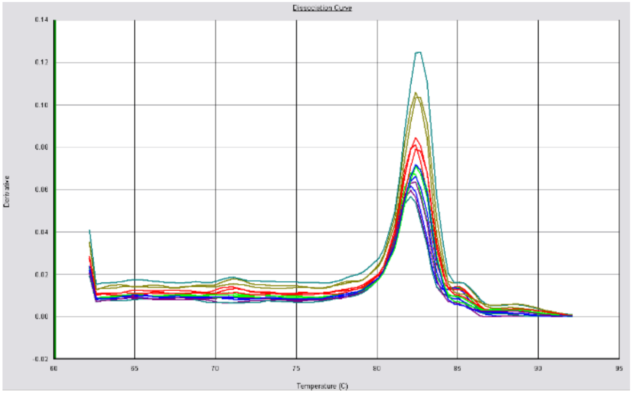

d) Ef1.2

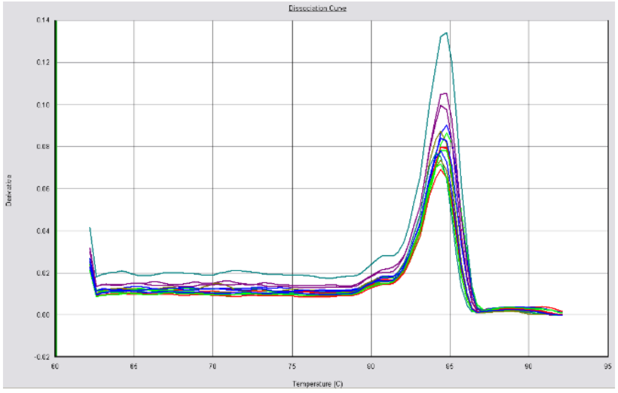

Supplement: Supplementary file 11 — Additional file 11: Fig. S3. Melting curves of some PCR products derived from the amplification of reference and target genes. [file 12870_2020_2616_MOESM11_ESM.pdf]
