# Supplementary material for: Transcriptome profiling at osmotic and ionic phases of salt stress response in bread wheat uncovers trait-specific candidate genes
Source: BMC Plant Biol. 2020 Sep 16;20:428. doi: 10.1186/s12870-020-02616-9 (PMC7493341; doi:10.1186/s12870-020-02616-9)
Supplement: Supplementary file 12 — Additional file 12: Fig. S4. Comparison of GFOLD values and ∆∆ Ct values of studied genes. [file 12870_2020_2616_MOESM12_ESM.pdf]

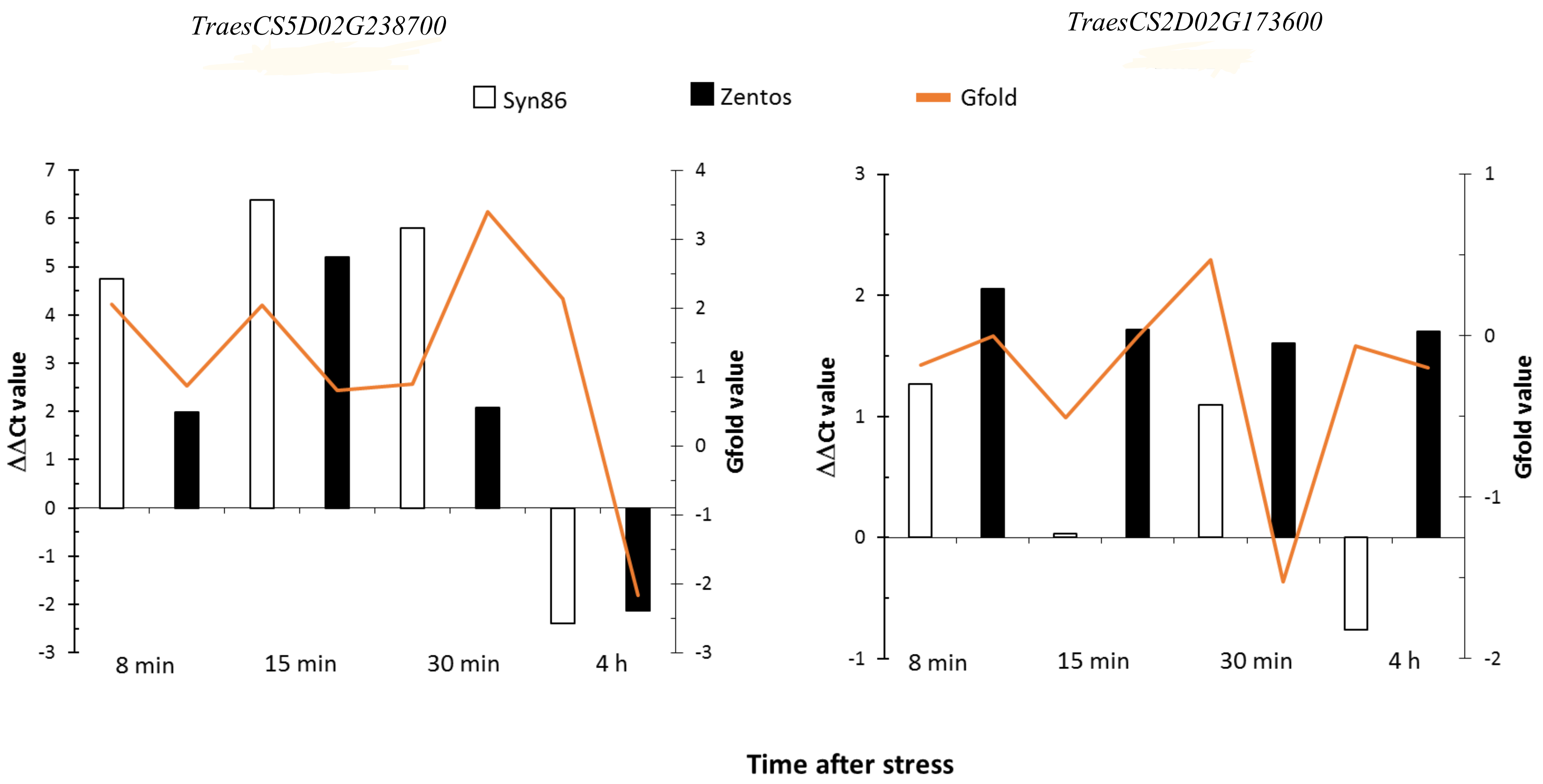

Fig. S4. Comparison of GFOLD values and ΔΔCt values of *TraesCS5D02G238700* and *TraesCS2D02G173600* in Syn86 and Zentos.
